# Cephalometric Analysis in Orthodontics Using Artificial Intelligence—A Comprehensive Review

**DOI:** 10.1155/2022/1880113

**Published:** 2022-06-16

**Authors:** Aravind Kumar Subramanian, Yong Chen, Abdullah Almalki, Gautham Sivamurthy, Dashrath Kafle

**Affiliations:** ^1^Department of Orthodontics, Saveetha Dental College and Hospital, Saveetha Institute of Medical and Technical Sciences, Saveetha University, Chennai 600077, India; ^2^Department of Stomatology, School of Medicine, Xiamen University, 361005, China; ^3^Orthodontics, Department of Preventive Dental Science, College of Dentistry, Majmaah University, Al Majmaah 11952, Saudi Arabia; ^4^Dundee Dental School & Hospital, University of Dundee, Scotland, UK; ^5^Department of Orthodontics, Kathmandu University School of Medical Sciences, Nepal

## Abstract

Artificial intelligence (AI) is a branch of science concerned with developing programs and computers that can gather data, reason about it, and then translate it into intelligent actions. AI is a broad area that includes reasoning, typical linguistic dispensation, machine learning, and planning. In the area of medicine and dentistry, machine learning is currently the most widely used AI application. This narrative review is aimed at giving an outline of cephalometric analysis in orthodontics using AI. Latest algorithms are developing rapidly, and computational resources are increasing, resulting in increased efficiency, accuracy, and reliability. Current techniques for completely automatic identification of cephalometric landmarks have considerably improved efficiency and growth prospects for their regular use. The primary considerations for effective orthodontic treatment are an accurate diagnosis, exceptional treatment planning, and good prognosis estimation. The main objective of the AI technique is to make dentists' work more precise and accurate. AI is increasingly being used in the area of orthodontic treatment. It has been evidenced to be a time-saving and reliable tool in many ways. AI is a promising tool for facilitating cephalometric tracing in routine clinical practice and analyzing large databases for research purposes.

## 1. Introduction

The various applications used regularly, such as Siri and Alexa, have been introduced due to the rapid rise in science and technology. Artificial intelligence (AI) and its aspects serve as the foundation for these applications. The term “artificial intelligence” is usually related to robotics. It describes using technology to create software or a piece of equipment that can easily imitate intelligence and accomplish tasks [1]. The phrase artificial intelligence (AI) refers to a discipline of research involved with designing programs and computers that can collect data, reason about it, and then transform it into intelligent actions [2].

John McCarthy invented the term AI in 1955, and he is widely regarded as the father of AI. John McCarthy coined the word to describe machines' ability to execute tasks that are classified as intelligent [3]. Machine learning algorithms are being used more frequently in orthodontics. Data mining, automated diagnostics, and landmark detection are some of the most often used applications now available [4]. The field of artificial intelligence includes an important branch called expert system (ES). The ES is an information and knowledge processing computer program system that consists mainly of a base of knowledge and an inferential machine. It imitates expert decision-making and work procedures while solving real-world problems in a single field [5]. AI allows for the organizing, investigation, categorization, and depiction of health data, and its influential design-obtaining and evaluated algorithms aid in the growth of science in general [6]. According to Morgan Stanley, the worldwide usage of AI in the medical sector might grow from $1.30 billion to $10 billion by 2024, a 40% annual growth rate [7].

Latest algorithms are developing rapidly, and computational resources are increasing, resulting in increased efficiency, accuracy, and reliability. Current techniques for completely automatic identification of cephalometric landmarks have established considerable efficiency improvements and increased prospects for their regular use [8, 9]. Deep learning, an advanced machine learning method, has recently gotten great attention. However, the primary move for implementing this newest technique to the automated system of cephalometric analysis was taken newly [10]. According to previous research, systems using the technique of random forest discovered 19 landmarks instantly. Computational performance is also essential when using automatic cephalometric in clinical practice, particularly when the procedure has to give out many landmarks to identify [9].

The primary considerations for effective orthodontic treatment are an accurate diagnosis, exceptional treatment planning, and good prognosis estimation. The AI technology has been used to determine if extractions are essential before the orthodontic treatment and the success of orthognathic surgeries [5, 11]. Arnett and Bergman stated in their article on face solutions in planning and diagnosis for orthodontic treatment that if the diagnosis is incorrect, the patient's aesthetics may deteriorate further, posing a considerable issue [12]. It implies that making diagnoses accurately by the dentist is an essential part of analyzing patients' problems. The main objective of the AI technique is to make dentists' work more precise and accurate. Image segmentation is vital in volumetric medical image analysis and automated or semiautomated computer-aided diagnosis systems. For decades, landmark identification in lateral cephalometric radiograph X-ray has been critical in diagnosis and treatment planning in orthodontic treatment [13]. Two hundred ninety-nine lateral cephalograms with 19 landmarks on *X* and *Y* coordinates were obtained from Colombian patients. The results showed that the selected mandibular variables were highly predictable and useful for craniofacial reconstruction [14].

Several studies investigated automated lateral cephalometric landmark identification [4, 15–18]. Arik et al. [8] used convolutional neural networks (CNN) to detect landmarks on lateral cephalogram automatically. Park and Hwang trained on 1028 cephalograms using the deep learning method. The transition from manual cephalometry to AI-based cephalogram is aimed at improving the diagnostic value of analysis by saving time and minimizing errors. Systematic and random errors are the most common types of errors in cephalometric analysis [19, 20]. A digital or scanned cephalometric image is saved in the database and added by software in automated cephalometric analysis. The identification of landmarks by software accomplishes the cephalometric dimensions automatically [21]. This narrative review is aimed at giving an outline of cephalometric analysis in orthodontics using AI.

## 2. Methodology

To select studies on AI in orthodontics, a narrative review was performed by utilizing Google Scholar, EMBASE, PubMed, MEDLINE, and Science Direct. An electronic literature search was conducted on August 10, 2021. The various search terminologies used were AI, machine learning, machine intelligence, deep learning, cognitive computing, radiomics, prediction machine cephalometric analysis, cephalometric prediction, cephalometric tracing, cephalometric landmarks, orthodontics, and dentistry. The literature search was restricted to the English language only and dated from 1980 to 2021. The significance of search results primarily assessed the articles depending on their abstract and title. After removing duplicate studies, two authors (AKS and YC) individually filtered the abstracts and titles of the citations obtained to eliminate nonqualified articles related to the study's qualifying keywords and criteria. The articles with abstracts or titles which are comprised of classified knowledge not relevant were excluded. The review included articles in the field of orthodontics that were AI-related. Only sufficient records of the data were used.

What about the reliability?

## 3. Results

The primary search strategy yielded 8420 records. About 135 articles were considered relevant to the reported electronic research. Seventy-one articles were omitted because they did not fulfill the eligibility criteria, and a manual reference search yielded no additional studies. Finally, the inclusion criteria resulted in 64 publications being considered for narrative review.

## 4. Discussion

### 4.1. History of AI in Orthodontics

The introduction of usage of AI in dentistry and orthodontics was to solve a variety of issues. The knowledge-based expert system was the first effort to use AI in the field of orthodontics and dentistry. These systems were designed primarily to assist dentists who were not specialists in developing proper diagnoses and successful treatment plans [22]. Research done by Alan Turing in 1950 began to investigate whether machines will have the same level of thinking ability as humans. Turing proposed a test that could be used to determine whether a machine is intelligent. The test is now regarded as a cornerstone of artificial intelligence. Professor Cahit Arf gave a talk at a conference in Turkey titled “Can Machines Think?” In 1958, he asked, “How does a machine think?” Professor Arf illustrated the idea of creating a machine that can think for itself, claiming that it is feasible [23].

After Alan Turing first proposed the “Turing test” to check the machine's ability to exhibit intelligent behaviour equivalent to human intelligence in 1950, John McCarthy provided the idea a name “Artificial Intelligence” in 1956. The development of stored-program electronic computers marks the beginning of modern AI. After seven decades, now we have an enormous set of AI-inspired applications, programs, and discoveries, with some of the most significant involvements coming from the orthodontics industry [20]. This discipline began when John McCarthy arranged a popular conference in 1956, an official AI-based research project. The conference ushered in a crucial period of AI research, which lasted from the 1950s to the 1970s [1].

**Figure 1:**
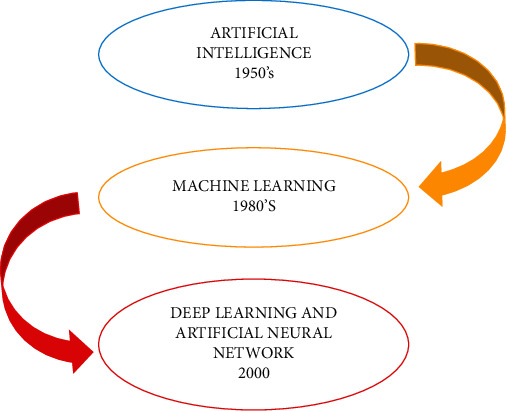
History of artificial intelligence.

Following the concept of artificial intelligence in the 1950s, the word “machine learning” was introduced in the 1980s, followed by the terms deep learning and artificial neural networks (Figure 1). To comprehend artificial intelligence, one must be familiar with terms such as machine learning, deep learning, and neural networks [23]. Cohen et al. made the first attempt at automated cephalogram landmarking in 1984 [24].

### 4.2. AI Used for Identification and Analysis of Cephalometric Landmarks

While artificial intelligence is essential in many fields, it is also becoming more prevalent in orthodontics. It has evolved into a valuable tool in orthodontics for correct diagnosis and proper management. The AI is primarily used to identify and analyze cephalometric landmarks, decision-making for tooth extraction, face analysis, tooth and mandible segmentation, bone age determination, prediction of orthognathic surgery, and temporomandibular bone segmentation [17, 18, 23]. Orthodontic diagnosis is a time-spending process that includes a dynamic examination of the patient, the review and analysis of photographs and radiographic recordings, and model analyses. Different treatment plans may emerge as a result of this complex assessment process among orthodontists. As a result, the orthodontic diagnosis must be automated to improve speed, consistency, and accuracy [25].

Despite advancements and successful implementation of AI in clinical settings, AI applications in dentistry have remained a rarity until now. The first favourable efforts at automated dental decay identification on intraoral radiographs were made [26, 27]. Since Broadbent and Hofrath invented the cephalometer in 1931, it has aided in the assessment of malocclusion and proven to be a reliable diagnostic tool in orthodontic practice and research [21]. The cephalometric radiograph assessment of sagittal and vertical skeletal structures introduced by Broadbent is used even now in orthodontic treatment planning. Cephalometric radiograph analysis relies on identifying radiographic landmarks and then measuring various distances, angles, and ratios [8]. The cephalometric analysis is mainly used for three reasons [23].

1. Depending on the available standards, a sagittal evaluation of hard and soft tissues in the head and face is performed
2. Changes identified during the reinforcement and treatment procedures
3. Development and growth as a factor in determining changes

**Figure 2:**
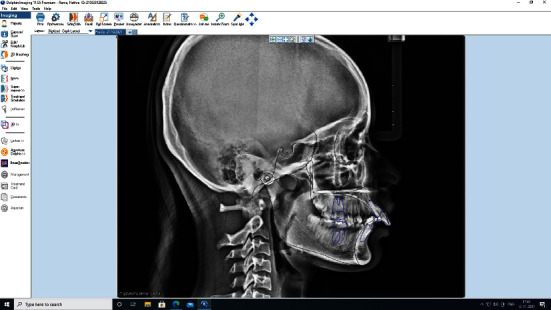
Cephalometric tracing done using Dolphin Imaging technology.

**Figure 3:**
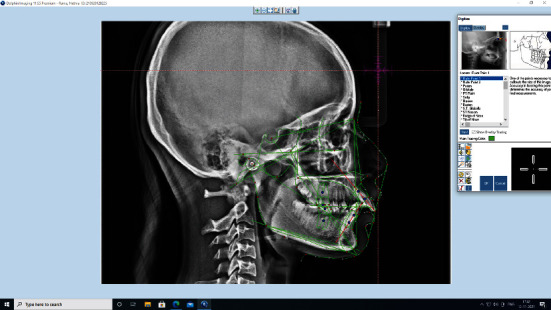
Prediction of cephalometric landmarks using artificial intelligence.

**Figure 4:**
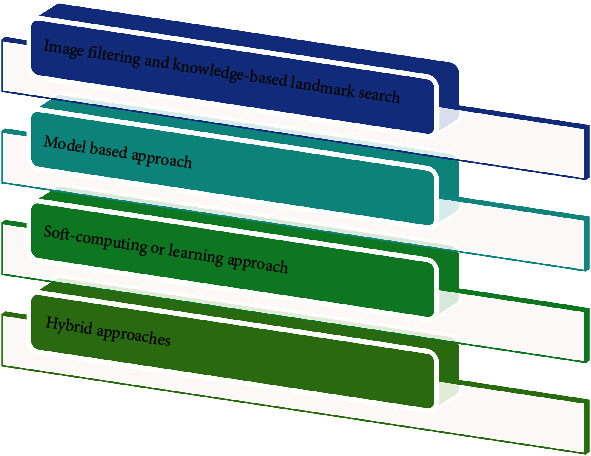
Artificial intelligence approaches to identify landmarks.

Manual tracing landmarks or AI approaches could be used to perform cephalometric analyses Figure 2. Manual tracing has been around for a long time and is a widely used method; moreover, it is time-consuming and liable to errors. Based on the orthodontists' experience, the cephalogram's quality, and several parameters to assess, manual tracing can take anywhere from 15-20 minutes to complete [26]. Upon tracing landmarks to be used in the design, automated cephalometric analysis transfers landmarks to a computer-attached digitizer; then, cephalometric analysis is completed via distances and angles measured by software after tracing landmarks. Artificial intelligence-assisted cephalometric studies minimize analysis time and enhance diagnostic value by reducing subjective errors [21, 28]. While the software is now generally used for cephalometric assessments, identifying the landmarks is still a routine task that requires the assistance of an orthodontic specialist. The level of quality of this analysis is primarily determined by the expert's experience [19]. Employing by means of improper identification of cephalometric landmarks can cause incorrect orthodontic treatment decisions, detecting completely automatic and accurate cephalometric landmarks is preferred, particularly for quality assurance [8]. This is where AI and machine learning can help orthodontists with their everyday activities. In a variety of ways, computer vision and AI techniques were used to detect cephalometric landmarks automatically Figure 3. Based on the methods used, or a combination of approaches, these approaches can be divided into four broad categories [21]. The approaches are explained in Figure 4.

### 4.3. Types of AI Programs or Software Used for Cephalometric Analysis

#### 4.3.1. Artificial Intelligence

While AI is a broad topic with many categories, from a computational standpoint, there are two major types: symbolic AI and machine learning. Symbolic AI refers to a set of methods for constructing algorithms in a way that is understandable to humans. This categorization, identified as “good old-fashioned AI” (GOFAI), was the research framework of AI till the late 1980s [29]. The different aspects of artificial intelligence used for cephalometric analysis are illustrated in Table 1.

#### 4.3.2. Machine Learning

The current paradigm is machine learning, a term coined by Arthur Samuel in 1952. The chief difference between symbolic AI and ML is that in ML, features acquire knowledge from explanations rather than from a system of rules devised by humans [6]. The purpose of machine learning is to make it simpler for machines to gain knowledge from records and find solutions without the assistance of humans. The most widely used ML techniques include the Bayesian Network classifier, verdict tree, reinforce path machine, random forest, logistic regression, fuzzified *k*-nearest neighbour, extreme learning machine, and convolution neural network [1, 30]. Depending on the algorithm's style of learning and the successful outcome, ML can be divided into three categories: organized learning (it is operated for prediction and classification based on an identified result), unorganized learning (it is used for finding designs and hidden configures with the unidentified result), and supported-learning (derived from previous versions, the machine creates a modified algorithm that enhances the intended remuneration) [31].

#### 4.3.3. Deep Learning

Deep learning (DL) is a type of machine learning in which a computer recognizes features in data. The DL's initial version is an artificial intelligence system, which was developed in the 1900s. As computational technology and power have increased exponentially, scientists have created more difficult and deeper neural network models to resolve more challenging and complex problems. DL is the new name for the neural network [7].

#### 4.3.4. Artificial Neural Network

The artificial neural network (ANN) is an algorithmic system that processes data in response to an external stimulus and is made up of artificial neurons, which are fully connected management elements. The artificial neuron is a simplified model that uses arithmetical structures to mimic the message assimilation and releasing behaviour of biological neural networks [32]. Artificial neurons are linked by interconnections that control data movement between them, just like their biological complements. Inhibitory or excitatory synapses or interconnections transmit stimulus from one processing element to another [33]. Neural networks have an advantage over traditional programmers. They can solve problems for which there is no computational solution or the existing solution is too complicated to find. The recognition and pattern prediction are examples of issues that ANNs are well suited to solving. ANNs have been used in the medical field for diagnosing, image and signal interpretation and analysis, and drug discovery [34, 35].

#### 4.3.5. Convolutional Neural Network

The convolutional neural network uses a DL system that can start taking a record picture and allocate significance to various aspects of it while also distinguishing between them. DL refers to CNN's ability to learn different aspects of an image or to be expected to handle the image's elegance better than regular classification algorithms [36]. The CNN's function is to compact the pictures into a template that is simpler to manage while still retaining essential details. With a deeper understanding of dentistry, CNN can create programs to detect pathologies, automatically identify cephalometric landmarks, segment teeth, and other structures [37].

#### 4.3.6. Planmeca Romexis Cephalometric Analysis Software

It allows for automatic cephalometric point detection and tracing in seconds; however, the software requires that a lateral radiograph be obtained only on the Planmeca cephalometric imaging unit, where it is automatically calibrated, resized, and oriented [38].

#### 4.3.7. YOLOv3 Algorithm

Redmon et al. [39] created the YOLO (You Only Look Once) family of CNNs for fast object detection, which was first described in the article “You Only Look Once: Truly united, Actual Object Recognition” published in the year 2015. The method is divided into three versions: YOLOv1, YOLOv2, and YOLOv3. The first version developed a general framework, the second version sophisticated the design, and the third version further enhanced the network model and training method. For automated cephalometric landmark identification in orthodontic clinical practice, YOLOv3 appeared to be more promising [4].

#### 4.3.8. Automatic Cephalon-Diagnostic Solutions

ACDS is an AI-based software that provides automatic cephalometric landmark detection, cephalometric tracing, measurements, and cephalometric analysis. After uploading thousands of cephalometric images to the computer database, the professor's group at Seoul National University Dental Hospital (SNUDH) developed the program. ACDS, according to the manufacturer, has a high level of accuracy in detecting cephalometric landmarks. Based on the evaluation of 80 landmarks in 253 consecutive digital lateral cephalometric radiographs, the error between the AI algorithm used in ACDS software and human examiners was 0.9 mm [40].

### 4.4. Web-Based Applications for Automated Cephalometric Analysis

The AI engine server performs the automatic cephalometric analysis. The user can also operate through the client webpage to correct the predicted landmarks. Operator information, cephalometric landmark locations, and cephalograms are all stored on the database server [41]. The types of web-based software are framed in Table 2 [42–45], and studies related to AI in the cephalometric analysis were summarised in Table 3 [4, 15, 19, 21, 32, 41, 46–63].

### 4.5. Conclusions and Future Perspectives

AI is increasingly being used in the area of orthodontic treatment. In many ways, it has been evidenced to be time-saving and a reliable tool. The AI is a promising tool for facilitating cephalometric tracing in routine clinical practice as well as analyzing large databases for research purposes. This review discusses the history, uses, and various methods of AI used for cephalometric assessment. The main objective of this narrative review was to assist clinicians and researchers in comprehending various features of this study area.

## Figures and Tables

**Table 1:** Types of artificial intelligence used for cephalometric analysis.

S/no.	Types of artificial intelligence
1	Machine learning (ML)
2	Deep learning (DL)
3	Artificial neural network (ANN)
4	Convolutional neural network (CNN)
5	Planmeca Romexis Cephalometric Analysis Software
6	YOLOv3 algorithm
7	Automatic cephalon-diagnostic solutions (ACDS)
8	Web-based applications for automated cephalometric analysis

**Table 2:** Type of web-based applications and software.

S/no.	Type of web-based applications	Reference
1	CephX (ORCA dental AI, Las Vegas, NV, an artificial intelligence-based software that performs automatic, instant cephalometric analyses	[42]
2	WebCeph and AutoCAD software	[43]
3	Dolphin Imaging, Dentofacial Planner, Quick Ceph, and FACAD	[46]
4	AudaxCeph and OrisCeph Rx	[45]

**Table 3:** Studies related to the application of AI for cephalometric analysis.

Authors and year	Aim	Number of X-rays
Grau et al., 2001 [49]	Aims to identify the landmarks on lateral cephalogram	20
Kim et al., 2020 [41]	The objective of this paper was to create a fully automated cephalometric analysis method based on deep learning, as well as a web-based application that did not require high-end equipment.	2075
Kunz, et al., 2020 [19, 29, 48]	The goal of this study was to use a specialized AI technique to compute an automatic cephalometric X-ray analysis.	1792
Ma et al., 2020 [56]	The goal of the research is to build a suitable automatic landmarking method depending on real OMS data to help surgeons save time during cephalometric analysis.	66
Mario et al., 2010 [33]	To provide an analysis of the cephalometric variables, taking into consideration the system's unspecific, inconstant, and paracomplete data	120
Neelapu et al., 2018 [54]	The study suggested a method for automatically identifying cephalometric landmarks depending on 3D CBCT image data.	30
Nishimoto et al., 2019 [15]	The objective of this research was to create deep learning based automatic cephalometric analysis technique for a computer using cephalogram pictures found online.	219
Rudolph et al., 1998 [53]	This study's goal was to create and test a new computer-based technique for automatically detecting cephalometric landmarks.	14
Rueda and Alcaniz 2006 [50]	The goal of this research is to develop an automated system that uses active appearance models (AAMs)	83
Tanikawa et al., 2010 [25, 52, 55]	The focus of this research was to assess the reliability of the system in recognizing anatomic landmarks and surrounding structures on lateral cephalograms using landmark-specific eligibility criteria.	65
Tanikawa et al., 2010 [25, 52, 55]	To evaluate the system performance that automatically identifies dentoskeletal characteristics on preadolescent children's cephalograms, and to develop a system to do so.	859
Vučinić et al., 2010 [49]	The objective of this study was to assess an automatic method for cephalogram landmarking that relied on an active appearance model (AAM), which is a statistical method that represents both shape and texture variations in the model's coverage areas by analyzing the form and grey-level appearance of an interest point.	60
Yu et al., 2020 [42, 47]	Final analytic methods employ a neural network in each process with lateral cephalograms to provide a reliable and accurate skeletal detection algorithm.	5890
Ed-Dhahraouy et al., 2018 [57]	The purpose of this study was to create a new method for automatically detecting points of reference in 3D cephalometry to overcome some of the limitations of 2D cephalometric analysis.	5
Muraev et al., 2020 [58]	The objective of this study was to create a machine learning technique capable of effectively placing cephalometric positions on frontal cephs and relating it to human accuracy.	300
Park et al., 2019 [4, 42, 43]	The goal of this research was to compare the accuracy and efficiency of two latest deep learning techniques for automatic cephalometric landmark identification.	1028
Hutton et al., 2000 [59]	The goal of this research was to see how precise the active shaped methodology was at locating cephalometric landmarks automatically.	5
Liu et al., 2000 [60]	The goal of this study was to see how precise an edge-based method could make a computerized automatic landmark detection system.	10
Yue et al., 2006 [61]	Aims to analyze all craniofacial anatomical structures.	200
Wang, C.-W., et al., 2016	The goal of the research was to look into and relate different techniques for automatically detecting landmarks in cephalometric X-ray images.	300
Hwang et al., 2021 [9, 61]	To compare a conventional cephalometric assessment with a fully automated cephalometric evaluation using the most advanced deep learning method for identifying cephalometric landmarks.	1983
Lee et al., 2020 [43. 66]	The goal of the study was to use Bayesian convolutional neural networks to create a new framework for finding cephalometric landmarks with competence areas (BCNN).	400
Jeon et al., 2021 [64]	The rapid development of artificial intelligence technologies for medical imaging has recently enabled the automatic identification of anatomical landmarks on radiographs. The purpose of this study was to compare the results of an automatic cephalometric analysis using a convolutional neural network with those obtained by a conventional cephalometric approach.	35
Leonardi et al., 2008 [21, 65]	To describe the techniques used for automatic landmarking of cephalograms, highlighting the strengths and weaknesses of each one and reviewing the percentage of success in locating each cephalometric point.	118 articles
